# Risky sexual practice and associated factors among people living with HIV/AIDS receiving antiretroviral therapy in Ethiopia: Systematic review and meta-analysis

**DOI:** 10.1371/journal.pone.0266884

**Published:** 2022-04-14

**Authors:** Habtamu Endashaw Hareru, Abdene Weya Kaso, Zemachu Ashuro, Moges Mareg

**Affiliations:** 1 School of Public Health, College of Health Science and Medicine, Dilla University, Dilla, Ethiopia; 2 Department of Environmental Health, College of Health Science and Medicine, Dilla University, Dilla, Ethiopia; 3 Department of Reproductive Health, College of Health Science and Medicine, Dilla University, Dilla, Ethiopia; The Technical University of Kenya, KENYA

## Abstract

**Background:**

The risky sexual behavior of people living with HIV/AIDS (PLWHA) may impose a risk of transmitting the disease to their partners and increase Human Immunodeficiency Virus (HIV) co-infection. This systematic review and meta-analysis aimed to determine the pooled prevalence of risky sexual behavior and associated factors among PLWHA receiving [Antiretroviral Therapy (ART)] in Ethiopia.

**Methods:**

To identify both published and unpublished research articles, systematic searches were performed in PubMed, HINARI, Medline, Science Direct, and Google Scholar databases. The review was carried out following the Preferred Reporting Items for Systematic reviews and Meta-Analyses (PRISMA) guideline. Cross-sectional studies reporting the prevalence of risky sexual practice and its associated factors among PLWHA receiving ART in Ethiopia were included. Two authors independently extracted all necessary data using a standardized data extraction format prepared in Microsoft Excel and exported to STATA version 14 statistical software for further analyses. The Cochrane Q test statistics and I^2^ test were used to assess the heterogeneity of the studies. Since the included studies exhibited considerable heterogeneity, the random-effects meta-analysis model was computed to estimate the pooled prevalence of risky sexual practice which was determined by dividing the total number of PLWHA with risky sexual practice practices by the total number of PLWHA on ART in the study and multiplied by 100. Furthermore, pooled odds ratio (OR) with 95% confidence interval (CI) was determined for the association between determinant factors and risky sexual practice.

**Result:**

In this study, 2351 articles were identified from different databases, and fifteen articles were selected for final systematic review and meta-analysis. In Ethiopia, the pooled prevalence of risky sexual practices was 43.56% (95% confidence interval (CI):35.51, 51.62). Discussion about safe sex with sexual partner/s [AOR = 0.26, 95% CI: 0.08, 0.92] and having multiple sexual partners [AOR = 1.90, 95% CI: 0.53, 6.84] were factors significantly associated with risky sexual practice in Ethiopia.

**Conclusion:**

A significant proportion of respondents engaged in risky sexual practices. Multiple sexual partners and a lack of discussion about safe sex are linked to a higher prevalence of the risky sexual practice in Ethiopia. It is critical to raise awareness about safe sexual practices during health education and counselling services and to encourage clients to freely discuss safer sex practices with their sexual partner/s at their antiretroviral therapy (ART) appointments as part of their follow-up care.

**Protocol registration:**

The protocol for this systematic review and meta-analysis was registered at PROSPERO (record **ID = CRD42021274600**, 25 September 2021).

## Introduction

Human Immunodeficiency Virus/ Acquired Immunodeficiency Syndrome(HIV/AIDS) is a major public health threat [1]. Globally, there are 37.7 million people were living with HIV, 1.5 million new cases, and 680 000 deaths due to AIDS-related illnesses, were reported yearly. Besides, around 27.5 million people had access to Antiretroviral Therapy (ART) [2]. Even though HIV prevalence is decreasing on a global scale, the majority of people with HIV were found in low- and middle-income countries. In 2020, over two-thirds of whom (25.4 million) were found in the World Health Organization (WHO) African Region [2]. Even though detailed research on the total economic cost of HIV/AIDS has not been conducted in the African region, studies conducted in other countries have found that HIV/AIDS has a statistically significant impact on Gross Domestic Product (GDP) [3, 4]. Moreover, it is heavily reliant on foreign donor aid to combat HIV issues in Sub-Saharan African countries [3].

In Ethiopia, 690,000 people were living with HIV in 2018, with an HIV prevalence of 1%. Besides, approximately 23,000 people were newly infected with HIV, and 11,000 died due to HIV/AIDS-related illness [5]. The HIV/AIDS epidemic in Ethiopia varies according to geographical location. The disease is seven times more prevalent in cities than in rural areas of the country. HIV/AIDS prevalence is greater than 1% in seven of the nine regional states and two city administrations. Gambela had the highest (4.8%) HIV/AIDS prevalence, followed by Addis Ababa (3.4%), Dire Dawa (2.5%), and Harari (2.4%) regional state [6].

The advancement of ART services has turned HIV/AIDS into a chronic illness that can be managed and has reduced HIV/AIDS morbidity and death. As a result, the majority of people on ART improve their physical condition and feel better. Individuals’ sexual desire and unprotected sexual practice may grow as a result of this enhancement [7, 8].

Risky sexual behavior in PLWHA includes having sex with multiple partners, using condoms irregularly, sharing needles and syringes for injecting medication, and having sex with partners who do not know (expose) their HIV status. PLWHA’s sexual risk behavior may threaten its partners with HIV or other Sexual Transmitted Infections (STIs). Such conduct can also lead to co-infection among HIV-infected individuals already [9]. Some studies have found that risky sexual behavior decreases after the start of ART, which could be attributed to serious counseling. However, many studies have found that risky sexual behavior increased after ART, which leads to worsening health outcomes, the harboring and spread of drug-resistant strains, posing a significant public threat, and increasing the risk of acquiring a sexually transmitted infection [8, 10–12].

The prevalence of risky sexual practices varies by country, ranging from 23% to 58% [8, 11, 13]. In Ethiopia, the risky sexual practices among PLWHA receiving ART ranged from 22% to 79.8% [7, 14–24], with the most prevalent factors associated with risk sexual practice being a lack of discussion about safe sex with their partner/s, an unknown partner’s serostatus, and having multiple sexual partners [16–18, 22, 24, 25].

Even though we discovered a remarkable variation in the prevalence of risky sexual practice among Ethiopian studies, methodological flaws associated with the measurement and significant inconsistency across the findings were observed. To the best of our knowledge, no previous systematic review and meta-analysis studies on the prevalence and associated factors of risky sexual behavior among PLWHA receiving ART were performed in Ethiopia. This had a significant impact for policy makers, program planners, and health service providers in the generation and dissemination of evidence-based information which necessitates designing and implementing appropriate interventions. Therefore, the objective of this systematic review and meta-analysis was to determine the pooled prevalence of risky sexual practices and associated factors among PLWHA receiving ART in Ethiopia.

## Methods and materials

### Protocol and registration

Study inclusion criteria and analysis were done according to the Preferred Reporting Items for Systematic Reviews and Meta-Analysis statement (The PRISMA 2020) guidelines [26]. The protocol for this systematic review and meta-analysis was registered at PROSPERO (record **ID = CRD42021274600**, 25 September 2021).

### Search strategy

To identify both published and unpublished research articles, systematic searches were performed on PubMed, HINARI, Medline, science direct, Google Scholar databases (Table 1), and Gray literature of observational studies were searched through the review of reference lists check and input of content experts. In addition, the Addis Ababa Digital Library was searched for unpublished papers relevant to this systematic review and meta-analysis. There were no restrictions imposed based on the year of publication. Endnote X7 software was used to retrieve and manage studies identified by our search strategy, as well as to remove duplicates. The literature search was carried out between the 5th of August and the 2nd of September and all papers published until the 2nd of September, 2021 were taken into account. The following search terms were used to find all relevant studies in the search databases: magnitude) OR prevalence) OR proportion) AND risky sexual practice) OR unsafe sex practice) OR Unprotected sexual practices) OR inconsistent condom use) OR no condom use) AND associated factors) OR risk factors) OR predictors) OR determinants) AND peoples living with HIV/AIDS) OR PLWHA) AND antiretroviral therapy) OR ART) OR highly active antiretroviral therapy) OR HAART) AND Ethiopia. We also viewed Science Direct and Medline using database-specific subject headings associated with the above keywords used in PubMed. The search terms were used both separately and in combination, using Boolean operators such as "OR" and "AND".

**Table 1:** Illustration depicting search results for the MEDLINE/ PubMed, Google Scholar, and other sources to determine the pooled prevalence of risky sexual practice and associated factors among PLWHA receiving (Antiretroviral Therapy) ART.

Databases	Searching key terms	Number of articles
**Google scholar**	"prevalence" or "magnitude" and "risky sexual practice" or "inconsistent condom use " and "determinants" or "associated factors" and "people living with HIV/AIDS" and "antiretroviral therapy" and “Ethiopia"	921
**MEDLINE/ PubMed**	("epidemiology"[Subheading]OR “epidemiology"[All Fields] OR “prevalence"[All Fields] OR "prevalence"[Mesh Terms]) AND "risky sexual practice"[All Fields] OR " unsafe sex practice "[MeSH Terms] OR "Unprotected sexual practices "[All Fields]) OR "inconsistent condom use"[All Fields]OR no condom use"[AllFields]) AND "associated factors" [All Fields] OR "risk factors"[MeSH Terms] OR "determinants" [All Fields] AND "People living with HIV/AIDS"[All Fields]AND PLWHA"[MeshTerms]AND"antiretroviral therapy"[All Fields] OR " ART "[All Fields] OR" highly active antiretroviral therapy" [All Fields] OR" HAART"[All Fields] AND ("Ethiopia"[MeSH Terms] OR "Ethiopia"[All Fields])	1137
**Other databases (sources)**	"prevalence" or "magnitude" and "risky sexual practice" or "inconsistent condom use " and "determinants" or "associated factors" and "people living with HIV/AIDS" and "antiretroviral therapy" and “Ethiopia"	293
**Total retrieved articles**		2351
**Full articles included in the systematic review and meta-analysis**		15

### Eligibility criteria

Two authors (HEH and ZA) independently screened the selected studies using their titles and abstracts for relevance to the review objective, and then retrieved the full text for further assessment. Disagreements were discussed during a consensus meeting with other reviewers (AWK and MM) for final selection of studies to be included in the systematic review and meta-analysis. Only studies conducted in Ethiopia, studies conducted on people living with HIV/AIDS received antiretroviral therapy, both published and unpublished, all observational study designs (i.e., cross-sectional, case-control and cohort) reporting the prevalence of the risky sexual practice and its associated factors, and reported in English language were included for this meta-analysis. However, studies that did not show clear data regarding the risky sexual practice, studies conducted on newly diagnosed HIV- positive individuals, articles without full- text (the exclusion of these articles is due to the inability to assess the quality of articles in the absence of full text., qualitative studies, editorials, and commentaries were excluded.

### Data extraction process

Two authors (HEH and ZA) independently extracted all the necessary data from the primary articles. The data were extracted using a standardized data extraction format prepared in Microsoft Excel in the form of summary table. Data extraction from each abstract and/or full text of the article considered eligible, information for the first outcome includes the titles, first author’s name, region, study area, publication year, study design, study setting, sample size, response rate, and the number of PLWHA with risky sexual practice with a 95% CI were recorded. For the second outcome (associated factors), data were retrieved in the form of two-by-two tables, and the log odds ratio for each factor was calculated based on the findings of the primary studies. The reviewers resolve disagreements through debate, and if they are unable to reach an agreement, other reviewers are consulted, and the disagreement is addressed by the full-text evaluation. When articles did not have adequate data, corresponding authors of the research articles were contacted using their email. HEH has been in charge of the entire data extraction and synthesis process.

### Outcome measurement

This review considered two main outcomes:—the first outcome was to determine the pooled prevalence of risky sexual practice among PLWHA receiving ART in Ethiopia which was determined as the total number of PLWHA with risky sexual practice divided by the total number of PLWHA on ART in the study and multiplied by 100. The second outcome was to determine factors associated with risky sexual practice), and the pooled odds ratios (OR) were computed by constructing two-by-two tables using the frequencies from primary studies for all associated factors.

### Operational definition

#### Risky sexual practice

In this review ‘‘risky sexual practice” mean that those articles reported risky sexual practice as non-use, inconsistent use, or inappropriate use of condom during sexual intercourse with either HIV-negative, positive or unknown sero-status partners before the data collection period.

### Quality assessment

The Newcastle-Ottawa Scale for cross-sectional studies quality assessment was adapted to assess the quality of included studies in this systematic review and meta-analysis [27]. Two authors (HEH & ZA) independently assessed the quality of the included studies. The problem of subjectivities between the two authors was solved through discussion and with the involvement of other authors (MM & AWK). The quality and eligibility of the screened articles were assessed using ten-star scores under four major categories; category I: **Selection (5 stars)**:- Representativeness of the sample (1star), sampling technique (1point), response rate (1 star), and ascertainment of exposure (2 stars); category II: **Comparability (2 stars)**:- confounding controlled (data/ results adjusted for relevant predictors/risk factors/confounders (2 stars); category III: **Outcome (3 stars)**:- Assessment of outcome (2 stars) and statistical tests (1stars). Finally, articles with a score of ≥ 6 out of 10 were considered high quality and included in the Meta-analysis.

### Data analysis

A Microsoft Excel spread sheet was used to extract the required data, which was then exported into STATA/SE version-14 statistical software for analysis. The I^2^ test statistic was used to determine the amount of heterogeneity between studies, which describes the percentage of total variation between studies that is due to heterogeneity rather than chance. An I^2^ statistic value of 25, 50, or 75% was used to indicate low, moderate, or high heterogeneity, respectively [28] and the presence of heterogeneity among reported prevalence was confirmed using Cochran’s Q test and P < 0.10 indicates statistically significant heterogeneity [29, 30]. As I^2^ statistic showed there is significant heterogeneity between studies (I^2^ = 98.1%, p <0.001), the pooled prevalence of risky sexual practice was determined using a random effect model. The random effect model is more conservative than the fixed effect model because it accounts for any heterogeneity in the meta-analysis. Subgroup analysis by publication year, sample size, and region of the study were done to decrease the random variations among the point estimates of original articles. Besides, for at least 10 included studies in the meta-analysis, the Egger weighted regression and Begg rank correlation test methods were used to statistically assess publication bias (two sided P–value of ≤ 0.05 was considered suggestive of statistically significant publication bias) and the forest plot was also used to graphically (visually) represent the presence of heterogeneity [31]. For the second outcome, pooled odds ratios (OR) with 95 percent confidence intervals (CI) for each factor were used to determine the relationship between risky sexual practice and its factors. Furthermore, Sensitivity analysis was used to see how a single study affected the overall estimate. Finally, tables and forest plots were employed to descriptively summarize the selected articles and findings.

## Results

### Study selection and identification

**Fig 1:**
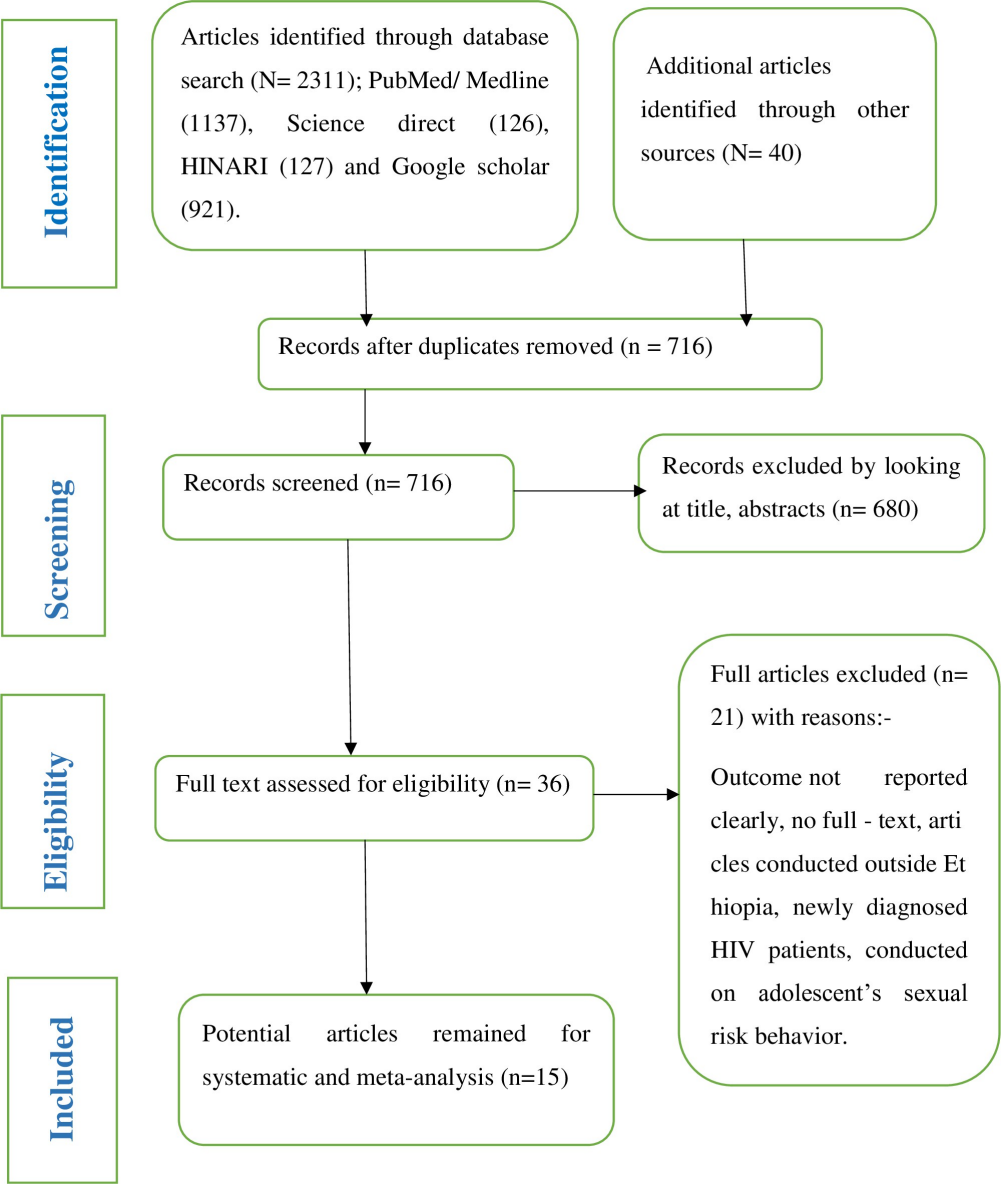
PRISMA flow diagram which shows the selection of articles for systematic review and meta-analysis.

A total of 2351 articles were found by searching the electronic databases (PubMed, HINARI, Medline EMBASE, and Google Scholar) and other sources. 1635 articles were removed due to duplication, 680 articles were removed after reading their titles and abstracts because they were irrelevant to this review, and 21 papers were removed due to unclear results and a lack of full text. Finally, 15 articles were included in this systematic review and meta-analysis, as shown in the PRISMA flow diagram (Fig 1).

### Characteristics of included studies

In this review, 15 relevant articles were included. The included studies were conducted between 2011 and 2021. All the included articles were utilized a cross-sectional study design and conducted at health institutions. Generally, a total number of 6913 participants 18 years and older were included for the current Meta—analysis. The lowest and highest sample sizes were 230 and 677, respectively [32, 33]. A minimum of (21.1%) and a maximum of (79.8%) prevalence of risky sexual practice were reported from the studies conducted in the Ahmara and Gambela region of Ethiopia, respectively [18, 34]. Six regions and one city administration (Addis Ababa) were represented for this review. Three from the Oromia region [7, 17, 25], four from Addis Ababa city administration [19, 22, 23, 33], three from South National and Nationalities of People’s Region (SNNPR) [16, 21, 24], four from Amhara region [14, 15, 32, 34] and one from Gambela region [19]. No studies were reviewed from Afar, Benishangul Gumez, and Harari and Tigray Regional states of Ethiopia. In terms of response rate, almost all studies had a high response rate (>91%), which may be due in part to the use of interviewer-administered questionnaires to collect data (Table 2).

**Table 2:** Characteristics of the included studies in meta-analysis based on publication year, region, sample size, response rate, and prevalence of the risky sexual practice in Ethiopia, 2021 (n = 15).

Authors	Publication Year	Region	Study area	Sample Size	Response rate	Prevalence
						(95% CI)
Abebo et al. [24]	2019	SNNPR	Arba Minch town	513	100%	52 (47.68,56.32)
Ali et al. [15]	2019	Amhara	Koladiba Health Center	394	91%	44.2 (39.06,46.34)
Anore et al. [16]	2021	SNNPR	Kembata Tembaro Zone	535	91%	40.9 (36.73,45.07)
Balis et al. [7]	2020	Oromia	West Wollega Zone	432	97.70%	56.9 (52.18,61.62)
Demissie et al. [19]	2015	Addis Ababa	Addis Ababa	376	100%	30.4 (25.75,35.05)
			(health centers)			
Dessie et al. [22]	2011	Addis Ababa	Addis Ababa	601	100%	36.9 (33.04,40.76)
			(hospital)			
Engdashet et al. [17]	2014	Oromia	Debrezeit Town	667	100%	22.2 (19.05,25.35)
Geleta et al. [33]	2020	Addis Ababa	Addis Ababa	677	100%	54.8 (51.0,58.55)
			( health centres)			
Molla et al. [14]	2017	Ahmara	Gondar University Referral hospital	518	99%	38 (33.80,42.20)
Mosisa et al. [25]	2018	Oromia	Nekemte Referral hospital	337	100%	32.9 (27.88,38.92)
Shewamene et al. [34]	2015	Amhara	University of Gondar Hospital	317	100%	21.1 (16.61,25.59)
Tadesse et al. [23]	2019	Addis Ababa	Addis Ababa	562	100%	39.1 (35.07,43.13)
			(hospital and health canter)			
Tesfaye et al. [21]	2020	SNNPR	Hawassa city	391	92%	48.6 (43.3,53.9)
Wendemagegn et al. [18]	2020	Gambela	Gambela town	360	97.80%	79.8 (75.61,83.99)
Yalew et al. [32]	2012	Amhara	Felege Hiwot Referral Hospital	233	98.7	56 (49.58,62.42)

### Quality of included study

We discovered that all of the included studies had reliable methodological quality during our quality assessment (NOS score ranges between 6 and 10 out of a total 10-point NOS score). The level of bias for the studies included in the final analysis was discovered to be moderate to the near-perfect agreement among investigators.

### Prevalence of risky sexual practice among PLWHA on ART

**Fig 2:**
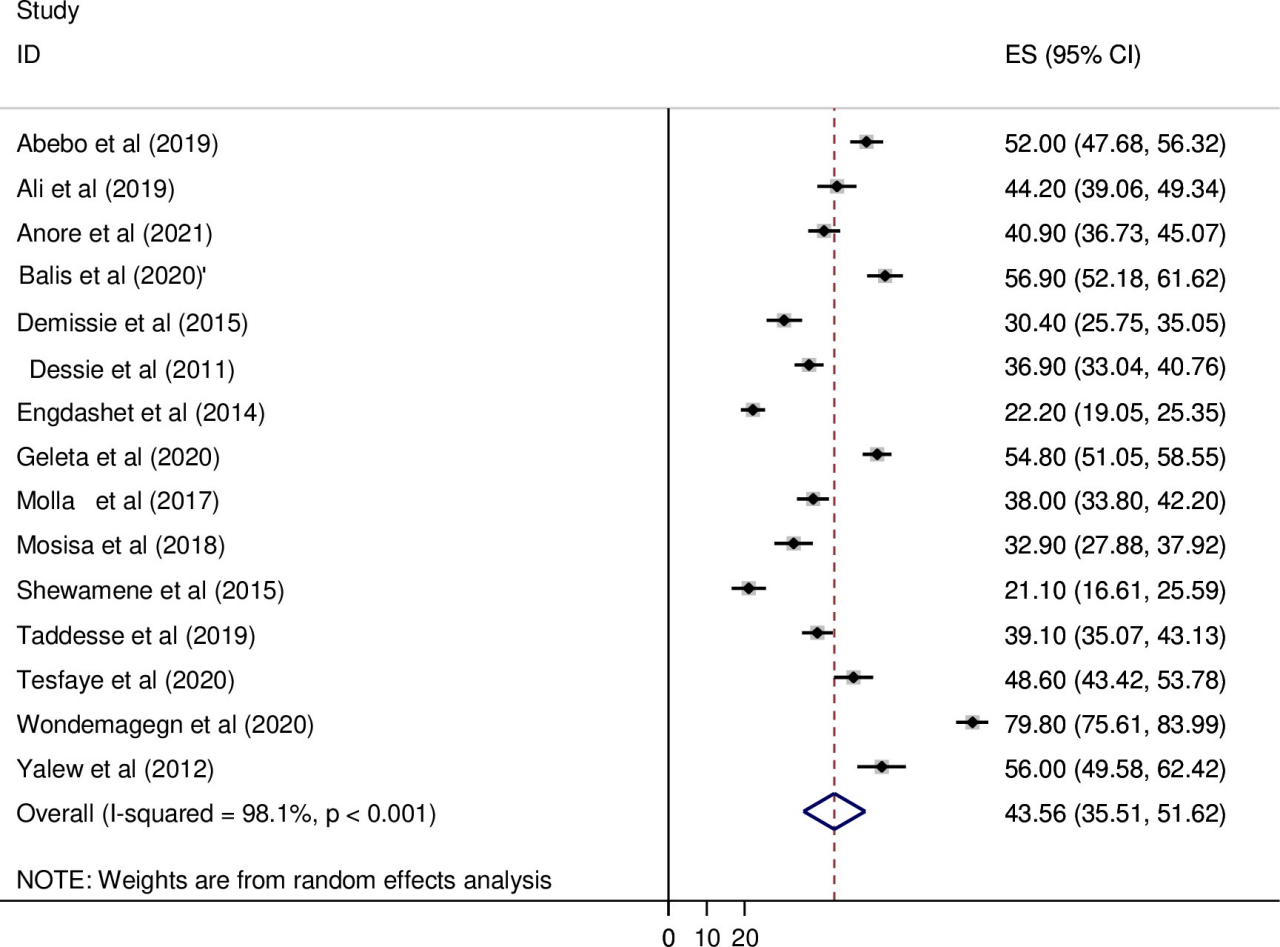
Forest plot revealing the overall pooled prevalence of the risky sexual practice in Ethiopia.

The overall pooled prevalence of the risky sexual practice in Ethiopia was 43.56% [95% CI: (35.51, 51.62)]. Significant heterogeneity among studies (I^2^ = 98.1%, p <0.001) was found, Due to this reason, the random-effects model was accustomed to estimate the Der Simonian and Laird overall effect (Fig 2).

### Subgroup analysis on the prevalence of the risky sexual practice

We had conducted subgroup analysis by study region, by publication year, and sample size. In the subgroup analysis, the highest prevalence of risky sexual practice was in South Nation Nationality Peoples Region (SNNPR) 47.12% (95% CI: 40.26, 53.98) and the lowest prevalence was observed in the Amhara region 39.69% (95% CI: 26.17, 53.23). We had observed significant heterogeneity among studies conducted in SNNPR (I^2^ = 85.4%, P < 0.001) and Amhara region (I^2^ = 96.7%, P< 0.001). However, a single study conducted in the Gambela region had the highest prevalence but we can’t consider it because of weak heterogeneity (I^2^ = 0.00). A Subgroup analysis was also conducted by years of publications, in which higher risky sexual practice was observed among studies published in the year 2017 and above 48.73% (95% CI: 40.22,57.26). The reported heterogeneity was significant for the year 2017 and above (I^2^ = 97.3%, p < 0.001) as well as for the year lower than 2017 (I^2^ = 96.5%, p < 0.001) years. Moreover, a subgroup analysis was also conducted by sample size revealed that the pooled prevalence of risky sexual intercourse was higher among studies having a sample size of less than 461, 46.23% (95% CI: 35.50,51,61) as compared to those having a sample size of 461 and above, 40.53% (95% CI: 39.76,49.37) (Table 3).

**Table 3:** Sub-group analysis of the prevalence of the risky sexual practice in Ethiopia based on random effect model, 2021 (n = 15).

Variables		Included studies	Sample Size	Estimates	Heterogeneity
				Prevalence (95% CI)	I^2^ (%), p- value
Region	Oromia	3	1,436	37.28 (16.54, 58.04)	98.6%, < 0.001
	Ahmara	4	1,462	39.69 (26.17, 53.23)	96.7%, < 0.001
	Addis Ababa	4	2,216	40.35 (30.09, 50.61)	96.1%, < 0.001
	SNNPR	3	1,439	47.12 (40.26, 53.98)	85.4%, < 0.001
	Gambela	1	360	79.18 (75.60, 83.99)	-
publication year	< 2017	5	2,194	33.11 (22.88, 43.33)	96.5%, < 0.001
	≥ 2017	10	4,719	48.74 (40.22, 57.26)	97.3%, < 0.001
Sample size	Less than 461	8	2,840	46.23 (35.50,51,61)	98.5%,< 0.001
	461 and above	7	4,073	40.53 (39.76,49.37)	97.2%,< 0.001

### Publication bias

**Fig 3:**
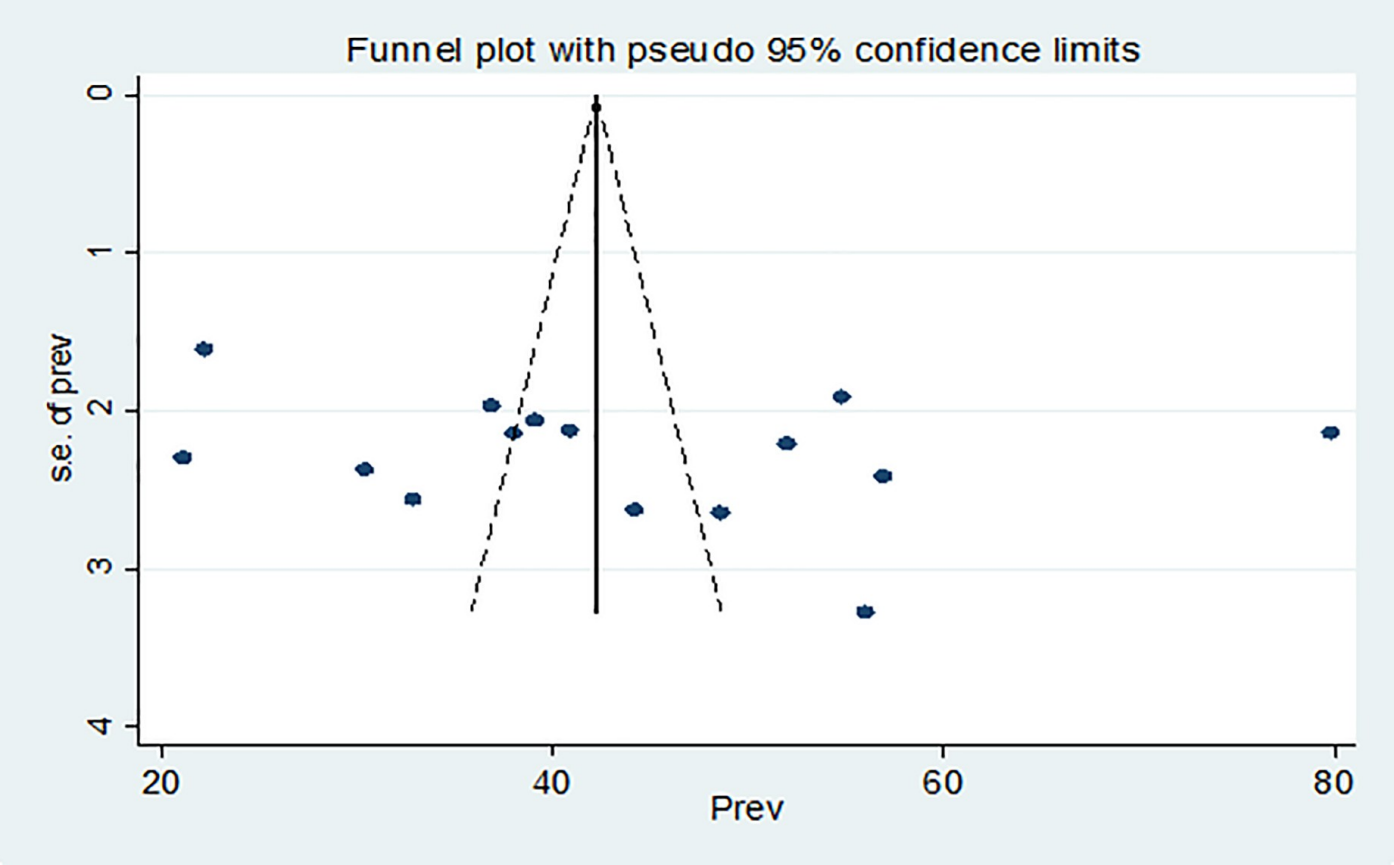
Funnel plot of publication bias for the pooled prevalence risky sexual practice of people living with HIV/AIDS on highly active antiretroviral therapy in Ethiopia, 2021.

To check publication bias among the included studies for the meta-analysis, Egger’s and Begg’s tests were carried out. Publication bias was not detected according to Egger’s test (β = 14.26, SE = 25.76, P = 0.291) and Begg’s (P = 0.488). Moreover, the shape of funnel plots shows the asymmetric distribution of the effect estimates (Fig 3).

**Fig 4:**
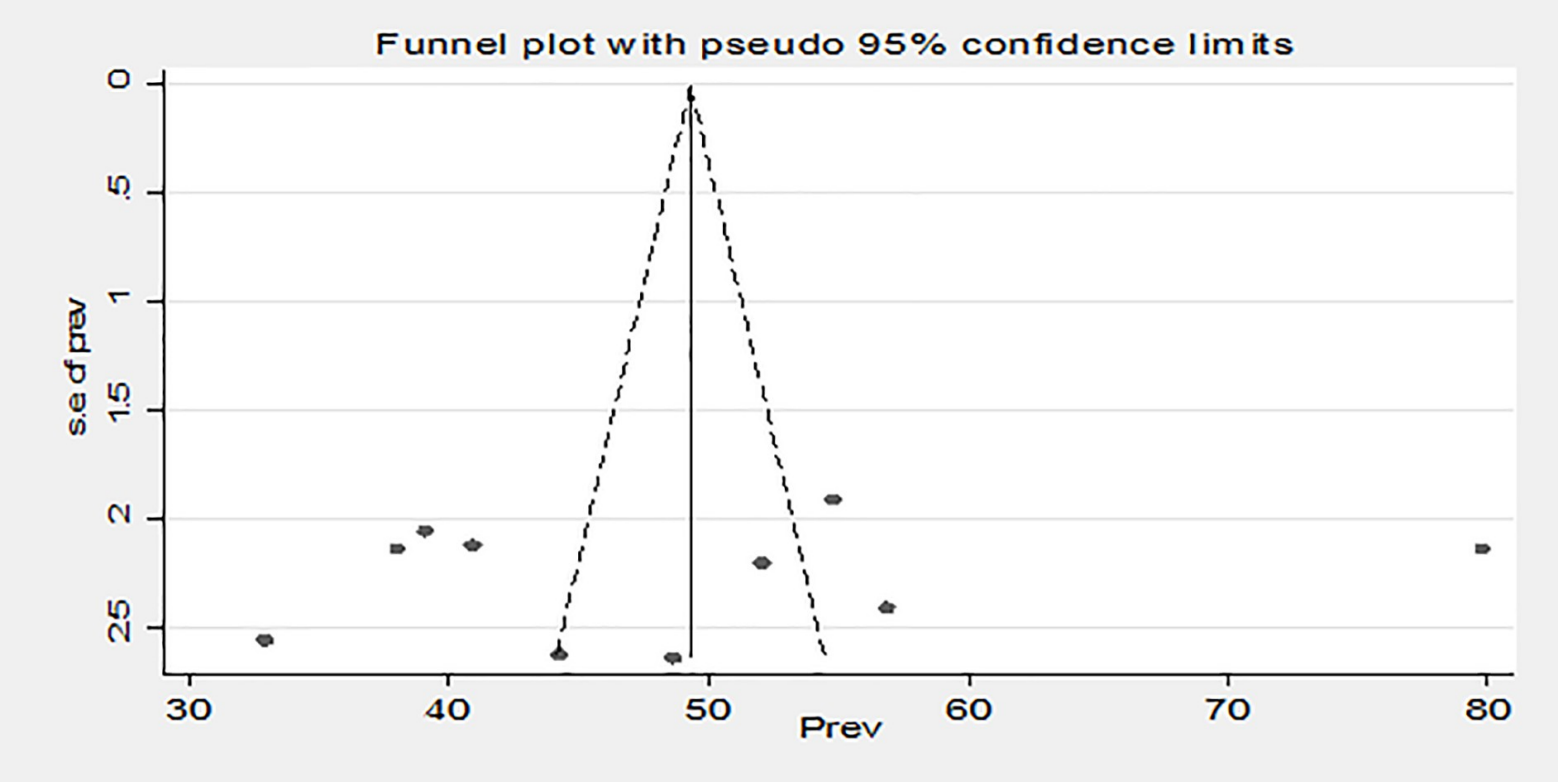
Funnel plot of publication bias for studies conducted in 2017 and above on the prevalence of risky sexual behavior among people living with HIV/AIDS on highly active antiretroviral therapy in Ethiopia, 2021.

Publication bias analysis was also conducted for the year 2017 and above, because the number of studies was at least ten. Therefore, Egger’s (p = 0.587) and Begg’s (p = 0.858) tests revealed that no publication bias was detected. Furthermore, the shape of funnel plots reveals the asymmetric distribution of effect estimates (Fig 4).

### Sensitivity analysis

**Fig 5:**
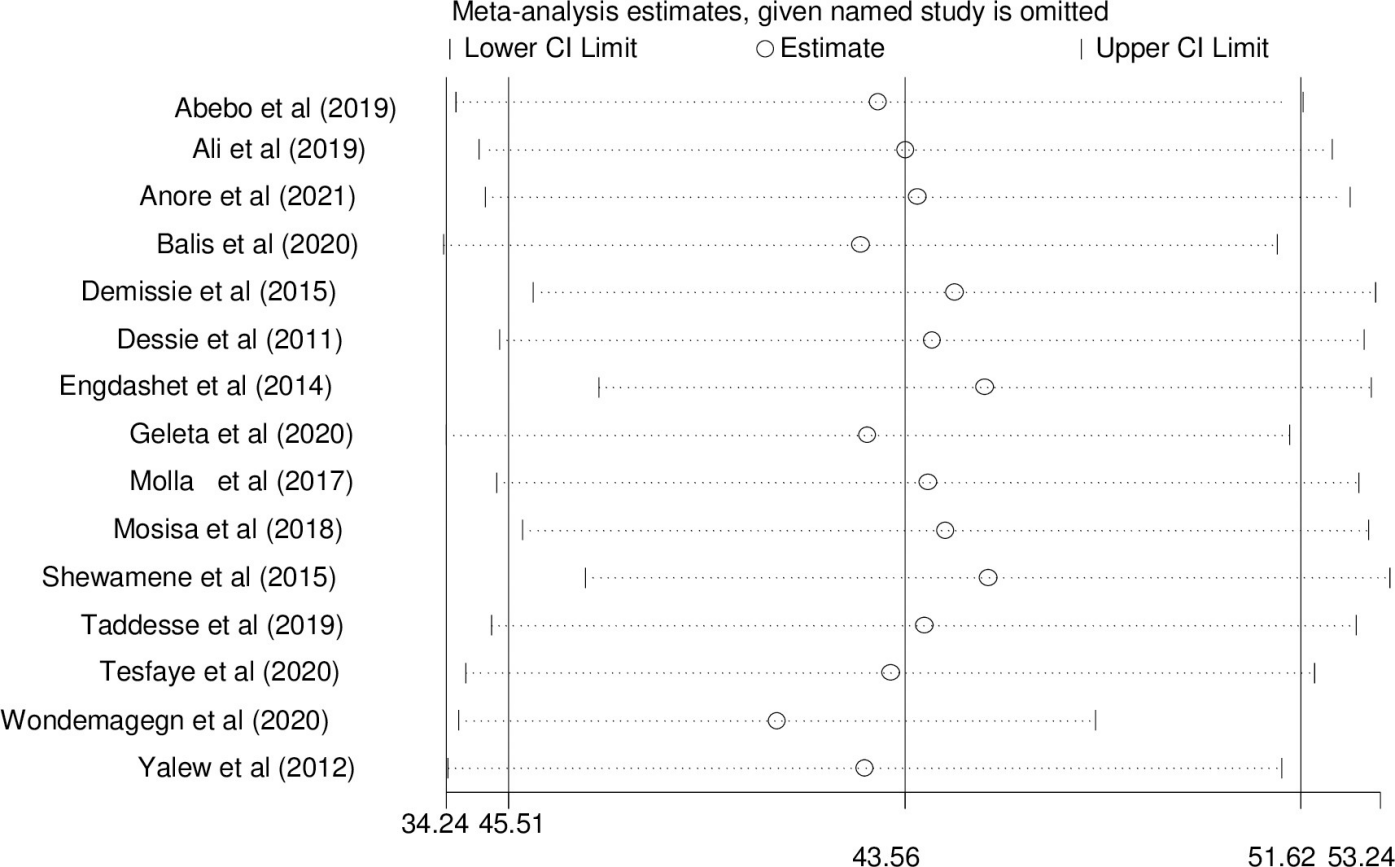
Sensitivity analysis of prevalence for each study being removed at a time: Prevalence and 95% confidence interval of risky sexual practice in Ethiopia.

We employed a sensitivity analysis to identify the potential source of heterogeneity in the analysis of the prevalence of the risky sexual practices in Ethiopia. The results of this sensitivity analysis showed that our findings were robust and not dependent on a single study (Fig 5).

### Factors associated with risky sexual practice

**Fig 6:**
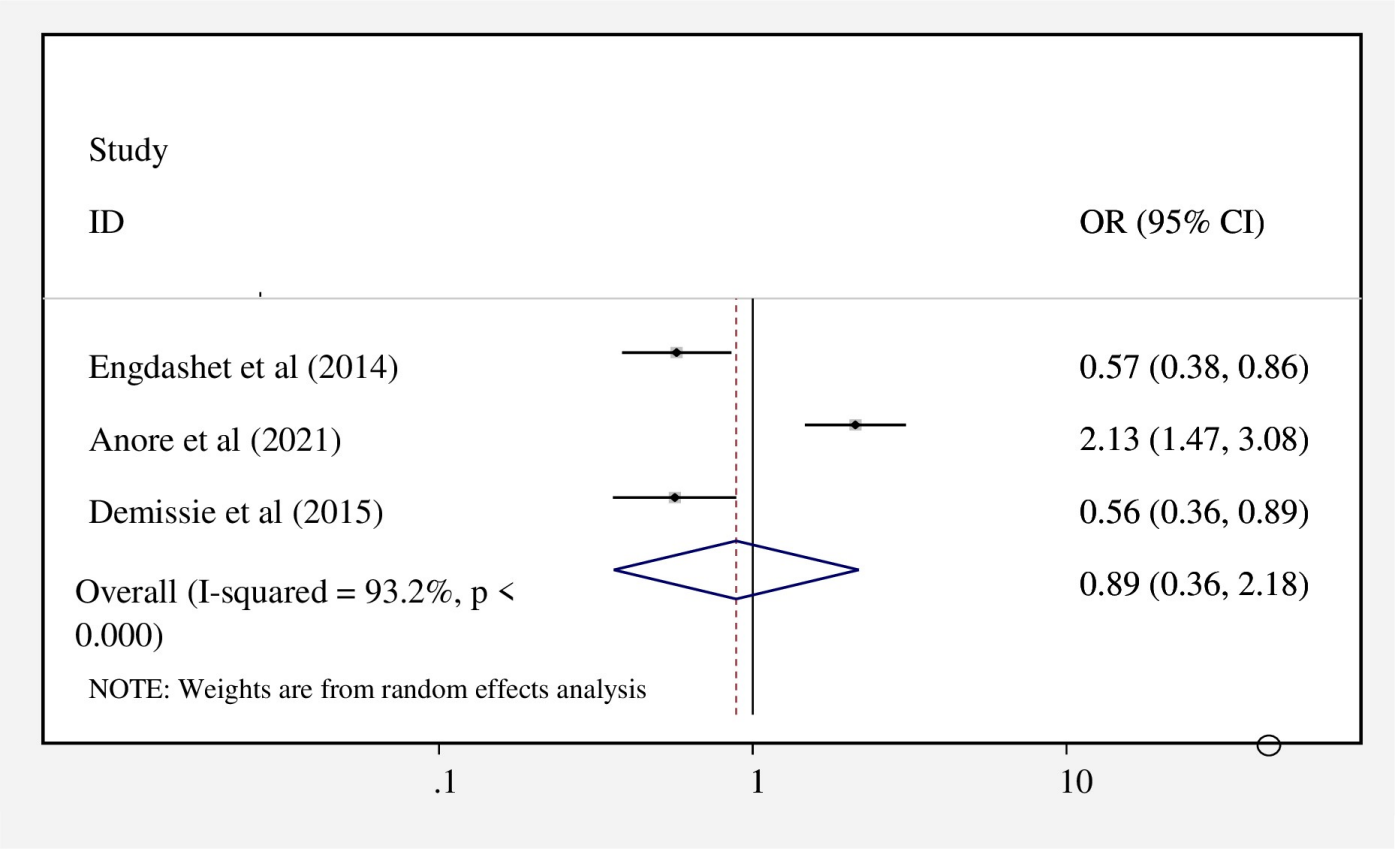
Forest plot showing the association between gender and risky sexual practice in Ethiopia.

Gender, discussion about safe sex with sexual partner/s, number of current partners (s), and unknown partner’s sero-status (HIV-status) were statistically significant factors for risky sexual practice in Ethiopia from previous primary studies, which we considered in this review. Three studies [16, 17, 19], were examined in this study to determine the relationship between risky sexual practices and gender. According to the findings of these three studies, there is no statistically significant association between risky sexual practices and gender [AOR = 0.89; 95%CI: 0.36–218] (Fig 6).

**Fig 7:**
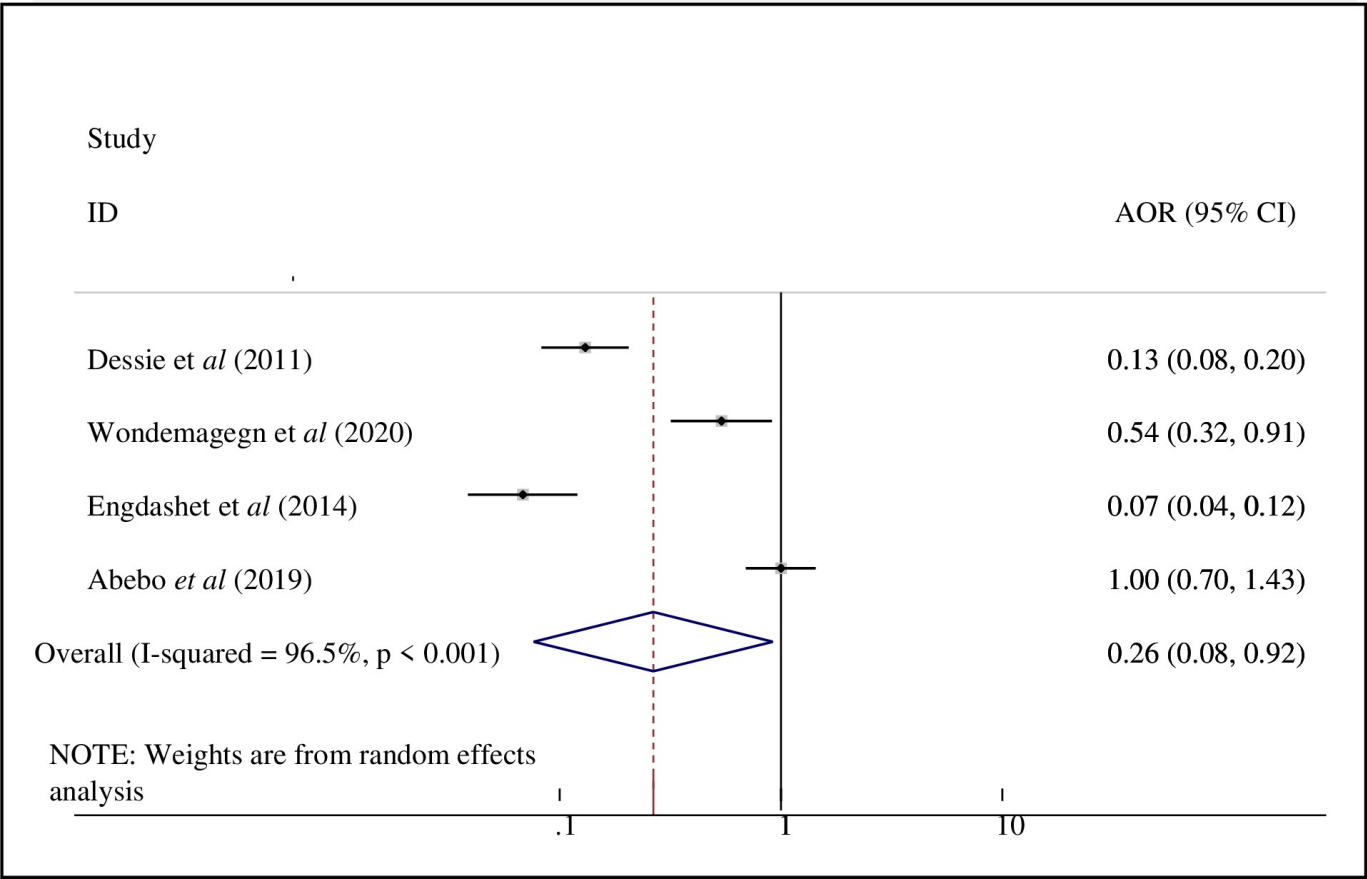
Forest plot of the association between discussion about safe sex with partner/s and risky sexual practice in Ethiopia.

Four studies [17, 18, 22, 24], were included to identify the association between Risky sexual practice and discussion about safe sex with their partner/s. From random-effects model estimate, the pooled odds of engaging in risky sexual practice among PLWHA who can discuss safe sex with partner/s were 74% less likely to engage in risky sexual practice than those who can’t discuss safe sex with partner/s [AOR = 0.26, 95% CI: (0.08, 0.92)]; with statistically significant heterogeneity between studies (I^2^ = 96.5%, p-value < 0.001) (Fig 7).

**Fig 8:**
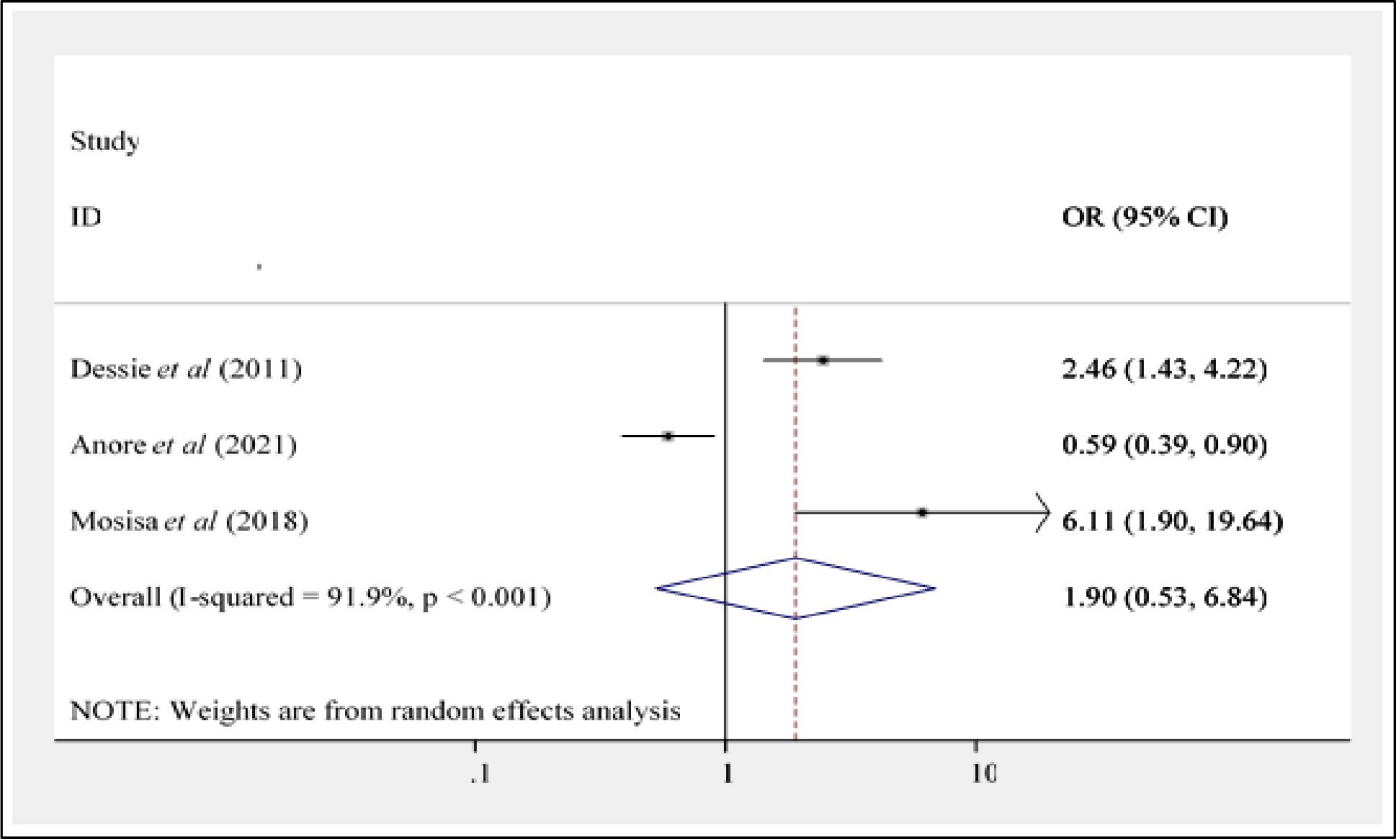
Forest plot showing the association between risky sexual practice and number of current partner(s) in Ethiopia.

Three studies [16, 22, 25] have been evaluated to check the presence of an association between risky sexual practice and the number of current partners (s). According to the findings of these three studies, there is no statistically significant association between risky sexual practices and gender [AOR = 1.90; 95%CI: 0.53, 6.84] (Fig 8).

**Fig 9:**
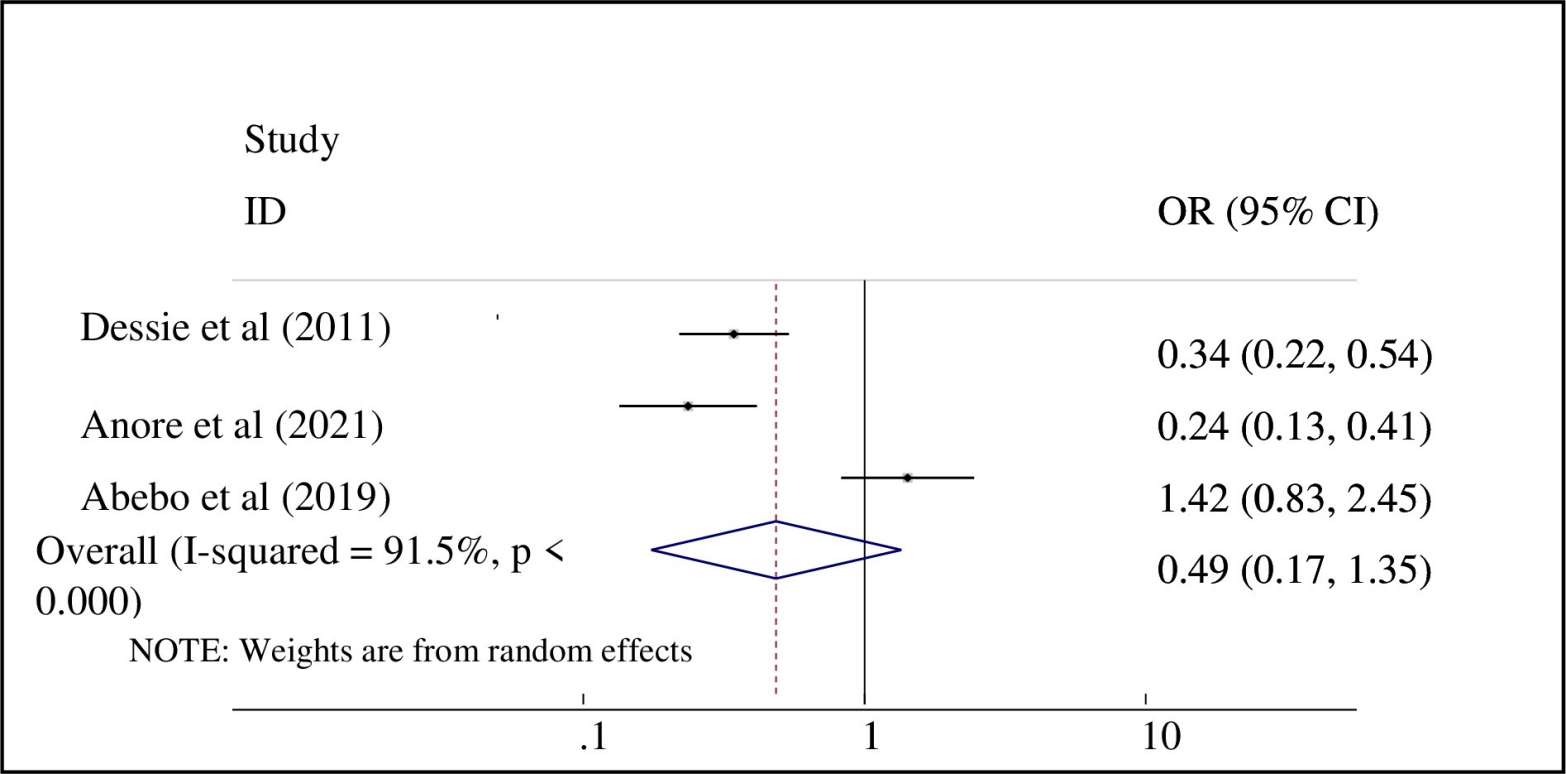
Forest plot showing the association between risky sexual practice and partner’s sero-status of PLWHA, Ethiopia.

Three primary studies were assessed that showed a significant association between risky sexual practice and partners sero-status [16, 22, 24]. In our meta-analysis, there was no statistically significant association between PLWHA and positive sexual partners (AOR = 0.49; 95% CI: 0.17, 1.35] (Fig 9).

## Discussion

In this systematic review and meta-analysis, we explored the prevalence of the risky sexual practices and their associated factors among PLWHA in Ethiopia.

In this meta-analysis, the pooled prevalence of the risky sexual practice in Ethiopia was 43.56% (95% CI: 35.51%, 51.62%). This finding is consistent with the study conducted in seven countries of Asian–pacific region reported 43% of PLWHA practiced inconsistent condom use at sexual intercourse with their regular partner [35], studies conducted in South Africa were 40.1% and 46.3% for males and women’s, respectively [8], studies conducted in Cameron and Rwanda reported 35.3% and 38% of PLWHA engage in risky sexual practice or use condom inconsistently, respectively [36, 37].

This finding was lower than a study conducted in the USA which reported 49.4% of PLWHA had unsafe sexual behavior, mainly inconsistent condom [38], in an India study in which Fifty-eight percentage of PLWHAs were practicing unsafe sex [11], a study conducted in Wakiso health center IV, Uganda (60.9%) [39] and a Nigerian study (56%) [40]. However, higher than study conducted in Jamaica, Brazil and Argentina, reported the prevalence of risky sexual practice or irregular condom use were 25%, 29% and 20%, respectively [41–43], and a study conducted in the United kingdom (UK) found that 12.8% of participants admitted to engaging in sexually risky behavior (unprotected sex in the previous 3 months with someone who was HIV-negative or of unknown HIV status) [44], and study in Togo (34.6%) [45], Nigeria (25.2%) [46], Uganda (25.9%) [47], and another a meta-analytic review conducted on Highly Active Antiretroviral Therapy and sexual risk behavior reported the median risky sexual practice among HAART attendees were 33% [48].

The main reasons for higher prevalence in the study setting might be due to misbelieve among PLWHA about the cessation of transmission of HIV after starting ART [11]; evidenced by a meta‑analytic review concludes that people’s beliefs about ART and viral load were found to be associated with increased risk of unprotected sexual intercourse [48], as more and more people with HIV believe that they live longer and healthier lives because of ART, an increasing number of sexual transmissions of HIV may arise from these PLWHA, desire for a child, and lack of self-efficacy in the condom, as well as having less awareness about HIV and believing that condom usage reduces enjoyment, may be related with an increased risk of unprotected sexual practice among PLWHA. Moreover, as evidenced by a systematic review and meta-analysis study on the correlates of unprotected sexual behavior among PLWHA, higher risky sexual practice may be associated with alcohol use [49], and non-disclosure to sexual partners may also result in continued unprotected sexual intercourse [50, 51].

Within the subgroup analysis by region, the South Nation Nationality Peoples Region (SNNPR) had the highest prevalence of risky sexual practice (47.12%, 95% CI: 40.26, 53.98), while the Amhara region had the lowest prevalence (39.69%, 95% CI: 26.17, 53.23). This could be related to differences in sample size, differences in evaluating risky sexual behavior, and poor health education for PLWHA during their ART follow-up. In terms of publication year, research published in 2017 and above had a greater risky sexual practice (48.73%, 95% CI: 40.22, 57.26) than studies published before to 2017 (33.11%, 95% CI: 22.88, 43.33). This could be attributed to the respondents’ improved health status following the introduction of ART, increased access to substance abuse/khat chewing, alcohol intake, smoking, the recent expansion of commercial sex workers, and negligence towards HIV prevention practice [52, 53]. As a result, experts in charge of illness prevention at this level should focus on PLWHA counselling, support, and awareness creation through various media such as radio, television, posters, leaflets, banners, conferences, conducting research, and the like in order to prevent the recent increase in risky sexual practice.

Males were shown to be less likely than females to participate in risky sexual behavior in this study. The link, however, was not statistically significant. In contrast, with the study conducted in seven countries of Asian–pacific region [35], a study conducted in Uganda [54] and Nigeria [46] found a strong link between gender and risky sexual behavior among PLWHA, with females being more likely to engage in risky sexual behavior. The result might be due to partner opposition being the most common reason for females not using condoms, poverty and persistent gender imbalances influence unprotected sexual behavior among women through a variety of mechanisms, including increased survival sex, low self-esteem, and inadequate condom bargaining skills, according to studies [55, 56]. As a result, interventions aimed towards HIV-infected females on ART may have a major influence on preventing HIV transmission in Ethiopia.

In our meta-analysis, PLWHA who had positive sexual partners were 51% less likely than those who had unknown/negative sexual partners to engage in risky sexual behavior. This result were consistent with a study conducted in France, which reported unprotected sex with a partner of negative or unknown HIV status would be more frequent among PLWHA [57] and prior studies conducted in South Africa and Nigeria, which found that mutual disclosure of HIV status was linked to consistent condom use, resulting in less unsafe sexual interactions [46, 58, 59] and. As a result, it is preferable to provide prevention and treatment programs that focus on encouraging the mutual disclosure of HIV status among PLWHA on ARV treatment, such as partner HIV counselling and testing. Moreover, these findings have implications for the development of interventions to reduce the risk of HIV transmission in Ethiopia.

The risky sexual practice was higher among PLWHA who had multiple sexual partners than with a single sexual partner. In line with studies conducted in the Brazil, USA, Vietnam, UK, and Nigeria which reported multiple sexual practices strongly related to risky sexual practice among PLWHA [38, 43, 44, 46, 60]. Therefore, it’s better to create awareness of the effect of sexual practice with multiple partners on HIV transmission risk and to implement continuous monitoring of the sexual experience of PLWHA during their follow–up visits.

Furthermore, PLWHA who discussion about safe sex with partner/s were 74% less likely to engage in risky sexual practices than those who had not discussed safe sex with their partner/. This finding was consistent with the study conducted in India [11]. This could be due to interpersonal communications regarding HIV transmission prevention, specifically whether sexual partners have explicitly discussed and agreed on the issue of safer sex. Furthermore, according to a meta-analytic review, counselling during HIV testing promotes safe discussion among clients, which leads to a decrease in sexually risky activities [61].

### Limitation of the study

This systematic review and meta-analysis has certain limitations. The study, only looked at the prevalence; however, because was all the articles included were cross-sectional studies rather than a longitudinal study, unable to look at the factors that lead to risky behavior. The small sample size of the selected articles could have potential influence on the pooled estimate, and they were all written in English. Furthermore, the studies looked at research conducted in a few Ethiopian regions, which may underrepresent the rest of the country.

## Conclusion and recommendation

A large percentage of respondents engaged in risky sexual practices. Multiple sexual partners and lack of discussion about safe sex are linked to a higher prevalence of the risky sexual practice, which could lead to re-infection with a new virus strain, other sexually transmitted illnesses, and HIV transmission. It is critical to provide health education and counselling that focuses on safe sexual practices at their ART appointments as part of their follow-up care. Besides that, it is preferable to provide them with the necessary information and support in order for them to adopt and maintain safer sexual practices. Furthermore, communication with the partner about safe sex should be encouraged, while an emphasis on need- based counselling is required at the programmatic level.

## Supporting information

S1 Checklist PRISMA (Preferred Reporting Items for Systematic review and Meta-Analysis) 2020 checklist: An updated guideline for reporting systematic reviews. (DOCX)

S1 File Quality assessment of studies using the modified Newcastle Ottawa scale for cross sectional studies for systematic review meta-analysis of risky sexual practice and associated factors among PLWHA receiving ART. (DOCX)

## Abbreviations

ART: Antiretroviral Therapy
AOR: Adjusted Odds Ratio
CI: Confidence Interval
WHO: World Health Organization
PLWHA: PLWHA: People Living with HIV/AIDS
HIV: Human Immune Virus
SNNPR: South National and Nationalities of People’s Region
