# Supplementary material for: Risky sexual practice and associated factors among people living with HIV/AIDS receiving antiretroviral therapy in Ethiopia: Systematic review and meta-analysis
Source: PLoS One. 2022 Apr 14;17(4):e0266884. doi: 10.1371/journal.pone.0266884 (PMC9009662; doi:10.1371/journal.pone.0266884)
Supplement: S1 File — (DOCX) [file pone.0266884.s002.docx]

(Supplementary 2). Quality assessment of studies using the modified Newcastle Ottawa scale for cross sectional studies for systematic review meta-analysis of risky sexual practice and associated factors among peoples living with HIV/AIDS Receiving Antiretroviral therapy.

|  | **Selection ( 5 stars)** | | | | **Comparability ( 2 stars)** | **Outcome ( 3 stars)** | |  |
| --- | --- | --- | --- | --- | --- | --- | --- | --- |
| **Study ID** | Representativeness of the sample(*) | Samples size(*) | Non- respondents(*) | Ascertainment of the exposure(**) | Confounding factors controlled(**) | Assessment of outcome(**) | Statistical test(*) | Total quality  score  (**10*)** |
| Abebo et al. | - | * | - | - | ** | ** | * | ******(6) |
| Ali et al. | * | * | * | - | ** | * | * | ******(*7) |
| Anore et al. | - | - | * | * | ** | ** | * | *******(7) |
| Balis et al. | * | * | * | - | ** | ** | * | ********(8) |
| Demissie et al. | * | * | - | ** | ** | ** | * | *********(9) |
| Dessie et al. | * | * | - | * | ** | * | * | *******(7) |
| Engdashet et al. | * | * | * | - | ** | ** | * | ********(8) |
| Geleta et al. | * | * | * | * | ** | * | * | ********(8) |
| Molla et al. | * | * | * | - | ** | * | * | *******(7) |
| Mosisa et al. | * | * | * | - | ** | * | * | *******(7) |
| Shewamene et al. | * | * | - | - | ** | * | * | ******(6) |
| Tadesse et al. | * | * | * | * | ** | ** | * | ********(8) |
| Tesfaye et al. | * | * | * | - | ** | * | * | *******(7) |
| Wendemagegn et al. | * | * | * | * | ** | ** | * | *********(9) |
| Yalew et al. | * | * | * | - | ** | * | * | ********(8) |
